# Musculoskeletal ultrasound findings in GCA with PMR and isolated PMR

**DOI:** 10.1093/rap/rkag110

**Published:** 2026-09-12

**Authors:** Soichiro Kubota, Nakako Tanaka-Mabuchi, Mayu Yazaki, Moe Nitta-Watanabe, Kojiro Ikeda, Ryosuke Ito, Yoshiaki Kobayashi, Kimie Harama, Kei Ito-Kobayashi, Shunichiro Hanai, Daiki Nakagomi

**Affiliations:** Department of Rheumatology, University of Yamanashi Hospital, Yamanashi, Japan; Department of Rheumatology, University of Yamanashi Hospital, Yamanashi, Japan; Department of Rheumatology, University of Yamanashi Hospital, Yamanashi, Japan; Department of Rheumatology, University of Yamanashi Hospital, Yamanashi, Japan; Department of Rheumatology, University of Yamanashi Hospital, Yamanashi, Japan; Department of Rheumatology, University of Yamanashi Hospital, Yamanashi, Japan; Department of Rheumatology, University of Yamanashi Hospital, Yamanashi, Japan; Department of Rheumatology, University of Yamanashi Hospital, Yamanashi, Japan; Department of Rheumatology, University of Yamanashi Hospital, Yamanashi, Japan; Department of Rheumatology, University of Yamanashi Hospital, Yamanashi, Japan; Department of Rheumatology, University of Yamanashi Hospital, Yamanashi, Japan

**Keywords:** PMR, GCA, musculoskeletal ultrasound, GCA–PMR spectrum disease, prediction model

## Abstract

**Objectives:**

To compare musculoskeletal ultrasound (MSUS)-defined musculoskeletal involvement between patients with isolated PMR and those with PMR symptoms in GCA and to explore the utility of MSUS findings combined with CRP levels for identifying GCA.

**Methods:**

We analysed 20 patients with GCA–PMR and 40 with PMR from a prospectively maintained MSUS registry before treatment initiation. Both shoulders and knees were assessed using greyscale (GS) and power Doppler (PD) ultrasound. Tenosynovitis, bursitis, tendinitis and ligamentitis were scored semiquantitatively. The association between ultrasound scores and serum CRP levels was evaluated. Exploratory multivariable regression analyses were performed to assess the potential utility of MSUS-based models in identifying GCA.

**Results:**

Across all assessed sites, the prevalence and severity of MSUS-defined inflammatory findings were consistently lower in the GCA–PMR group than in the PMR group. Total GS and PD scores were significantly lower in the GCA–PMR group, despite comparable CRP levels between the groups. A model combining CRP levels and total GS+PD scores yielded an area under the curve (AUC) of 0.94 for identifying GCA. A similar performance was observed with simplified models based on selected anatomical sites.

**Conclusions:**

Patients with PMR symptoms in GCA exhibited lower MSUS-defined musculoskeletal involvement than those with isolated PMR. These findings suggest that reduced musculoskeletal inflammatory burden relative to systemic inflammation may be a characteristic feature of patients with GCA presenting with PMR symptoms. MSUS findings combined with CRP levels may provide complementary information when evaluating patients with PMR for possible GCA.

## Graphical abstract

**Graphical Abstract:**
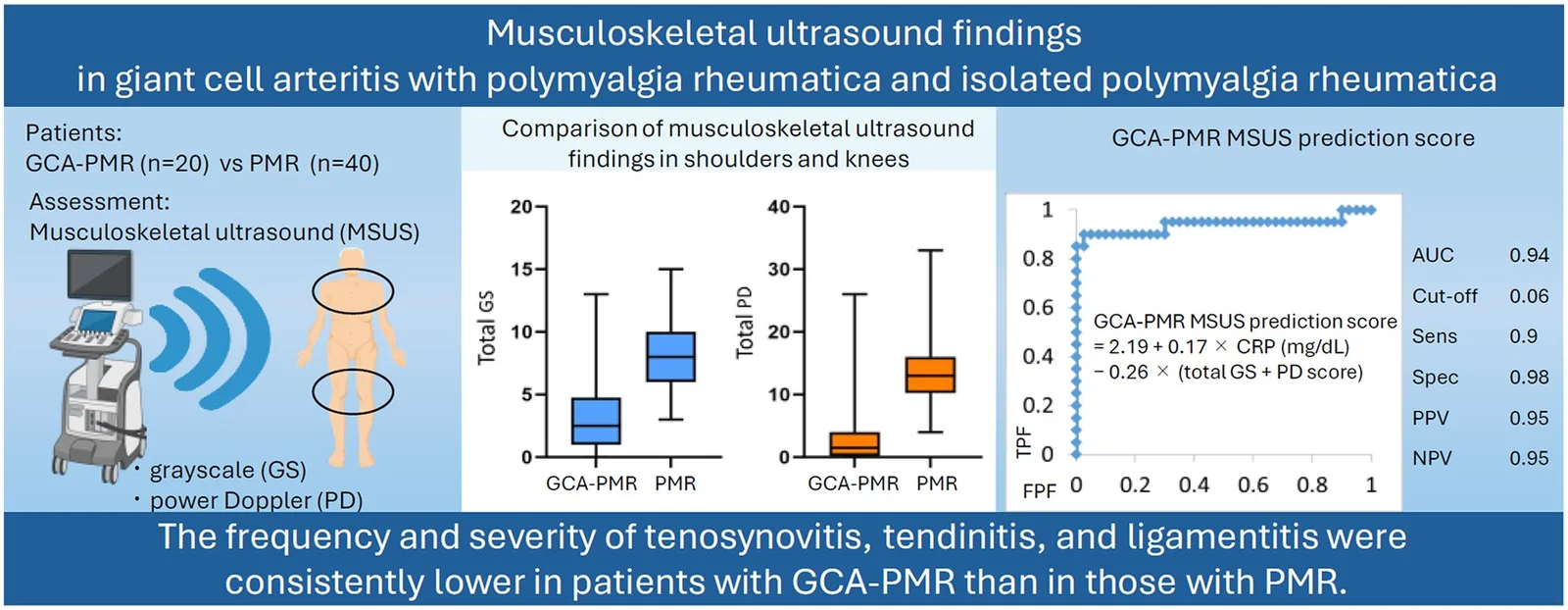

Key messages

- Patients with GCA–PMR exhibit less ultrasound-defined musculoskeletal involvement than those with isolated PMR.
- CRP levels and musculoskeletal ultrasound findings are discordant in GCA–PMR.
- Combined CRP and musculoskeletal ultrasound findings may identify GCA in patients with PMR.

## Introduction

GCA is a large-vessel vasculitis (LVV) that affects people aged 50 years or older and is characterized by the involvement of cranial and extracranial arteries. Patients with GCA frequently have PMR symptoms, which typically present with morning pain and stiffness in the shoulders, neck and hip girdle [1, 2]. Traditionally, the diagnosis of PMR is based on clinical signs and symptoms, and high levels of inflammatory markers on laboratory testing. However, in recent years, imaging modalities such as musculoskeletal ultrasound (MSUS), MRI and PET/CT have been shown to improve diagnostic accuracy [3]. In particular, the 2012 EULAR/ACR provisional classification criteria for PMR incorporate MSUS inflammatory findings of the shoulder and hip joints to support PMR classification [4]. Although the addition of MSUS to the provisional classification criteria provides only a modest improvement in classification performance, MSUS remains valuable for identifying characteristic inflammatory lesions in PMR [5]. MSUS studies of the shoulder and knee joints in PMR demonstrated that characteristic PMR lesions are mainly periarticular, such as bursitis, tenosynovitis, tendinitis and ligamentitis, rather than intra-articular [6, 7].

Systematic comparisons of musculoskeletal involvement between isolated PMR and PMR symptoms in GCA are limited. Although several studies have evaluated the shoulder and hip joints by using MSUS, MRI or PET-CT, the examined sites and semi-quantitative scoring methods have not been standardized, and assessments have generally been limited to the presence or absence of inflammatory findings rather than a detailed characterization of musculoskeletal involvement [8–11]. Consequently, no study has comprehensively compared the patterns of musculoskeletal involvement between patients with isolated PMR and those with PMR symptoms in GCA, leading to uncertainty regarding the potential differences in musculoskeletal manifestations. The frequency, severity and anatomical distribution of musculoskeletal involvement in isolated PMR and PMR symptoms in GCA remain unclear, and no framework has been established for assessing disease activity in patients with GCA–PMR.

In this study, MSUS was used to compare musculoskeletal involvement in patients with isolated PMR or PMR symptoms in GCA.

## Methods

### Patients

We recruited patients with GCA presenting with PMR symptoms who underwent MSUS at the University of Yamanashi Hospital between 2016 and 2024. Patient characteristics and MSUS data were obtained from a prospectively maintained MSUS registry of patients with PMR at our institution [6]. Patients included in this registry were evaluated before the initiation of any therapeutic intervention. Clinical data and MSUS findings were collected before treatment, and none of the patients received glucocorticoids (GCs) or non-GC immunosuppressive agents. GCA and PMR were diagnosed by expert rheumatologists at the time of presentation based on the overall clinical assessment and the available diagnostic investigations. For the present study, all patients were subsequently evaluated using the 2022 ACR/EULAR classification criteria for GCA [12], and the 2012 EULAR/ACR provisional classification criteria for PMR [4], and only those fulfilling the respective criteria were included. Patients were excluded if they did not meet the applicable classification criteria or had a history of treatment with GC or non-GC immunosuppressive agents. Among patients with PMR in the registry, 20 consecutive patients diagnosed with GCA (hereafter referred to as the GCA–PMR group) were included. For comparison, 40 patients with isolated PMR (hereafter referred to as the PMR group) were randomly selected from the same MSUS registry. The patient selection process is shown in Fig. S1, available at *Rheumatology Advances in Practice* Online. All patients were followed for at least 12 months after diagnosis. The final diagnosis was reviewed during follow-up by expert rheumatologists, and no diagnostic reclassification occurred in either group.

### Clinical assessment

Before treatment initiation, the demographic characteristics and clinical items in the 2022 ACR/EULAR classification criteria for GCA and the 2012 EULAR/ACR provisional classification criteria for PMR were assessed in all enrolled patients [4, 12].

### MSUS assessment

MSUS examinations were performed by rheumatologists certified in MSUS at the Japan College of Rheumatology (JCR). MSUS assessments were conducted using either a HITACHI ARIETTA 60 or HI VISION Preirus equipped with a multi-frequency superficial linear probe (5–18 MHz) (Hitachi Medical Corporation, Tokyo, Japan). The standardized MSUS settings recommended for each device were used. Bilateral shoulders and knees were scanned in accordance with previously reported MSUS protocols for PMR [6]. The hip joints were not included in the predefined registry protocol. The ultrasound assessment focused on shoulder and knee lesions because these sites had been systematically evaluated in our previous PMR cohort and were considered feasible for a standardized assessment in routine clinical practice. For the shoulder, the long head of the biceps tendon (LHBT), subacromial/subdeltoid bursa (SASDB), supraspinatus tendon (SSpT), subscapularis tendon (SScT) and glenohumeral joint (GHJ) were evaluated. For the knee, the suprapatellar bursa (SPB), medial and lateral aspects of the knee joint, medial and lateral collateral ligaments (MCL and LCL), and popliteus tendon (PopT) were assessed. MSUS images were evaluated for synovitis, bursitis, tenosynovitis, tendinitis and ligamentitis.

Tenosynovitis was evaluated in the LHBT and PopT. Tendinitis was assessed in the SSpT and SScT, and ligamentitis in the MCL and LCL. Knee synovitis was assessed at the medial and lateral parapatellar recesses. The patellar tendon and superficial or deep infrapatellar bursae were not included in the predefined registry protocol. Synovitis, tenosynovitis and bursitis were semi-quantitatively graded for greyscale (GS) abnormalities and power Doppler (PD) signals by using a 4-point scale (0–3), as previously described [6, 13–16]. For tendinitis and ligamentitis, only PD signals were graded on a scale of 0–3, according to previous reports. Tendinitis and ligamentitis were defined as the presence of PD activity within or adjacent to the tendon or ligament. GS assessment was not performed for tendons or ligaments because age-related degenerative changes, including thickening and structural abnormalities, are common in elderly individuals and may not reliably reflect active inflammatory involvement in this population. Therefore, PD activity was used as the principal marker of inflammation at these sites. All MSUS findings were determined by consensus among three JCR-certified rheumatologists. During the image evaluation, the assessors were blinded to the patients’ identities and clinical information.

The criteria for defining MSUS inflammatory findings were based on previous reports: synovitis, tenosynovitis and bursitis were considered positive when the GS score was ≥2 or the PD score was ≥1, whereas tendinitis and ligamentitis were considered positive when the PD score was ≥1 [6]. For quantitative analysis, the total GS and PD scores were calculated as the sum of the semi-quantitative GS (0–3) and PD (0–3) across all evaluated sites. The total GS score was defined as the sum of GS scores across seven assessed sites bilaterally (14 sites in total), excluding tendons (SSpT and SScT) and collateral ligaments (MCL and LCL), for which GS assessment was not performed; the maximum possible score was 42 points. The total PD score was defined as the sum of the PD scores across all 11 bilaterally assessed sites (22 sites in total), with a maximum possible score of 66.

### Statistical analyses

Statistical analyses were performed using SPSS software (version 27.0; IBM Japan, Tokyo, Japan). The normality of continuous variables was assessed using the Shapiro–Wilk test. Normally distributed continuous variables were summarized as mean (standard deviation) and compared using parametric tests (Student’s t-test). Non-normally distributed continuous variables were summarized as medians (interquartile range) and compared using nonparametric tests (Mann–Whitney U test). Categorical variables were summarized as percentages and compared using the χ² or Fisher’s exact test, as appropriate. To develop a predictive model for GCA in patients with PMR, multivariable binary logistic regression analysis was performed using the forced-entry method. The presence or absence of GCA in patients with PMR was used as the dependent variable, and CRP and MSUS scores were included as independent variables. The following MSUS score combinations, which were calculated as the sum of the left- and right-sided grading scores, were evaluated as candidate predictors.

1. Total GS score + total PD score
2. GS score + PD score in bilateral LHBT
3. GS score + PD score in bilateral LHBT, SSpT and SScT
4. GS score + PD score in bilateral LHBT and PopT

For each model, the sensitivity, specificity, positive predictive value and negative predictive value were calculated, and receiver operating characteristic curve analysis was performed to determine the area under the curve (AUC) and optimal cutoff value. All statistical tests were two-sided, and *P* < 0.05 was considered statistically significant.

### Ethics approval and consent to participate

This study was approved by the Ethics Committee of the University of Yamanashi (approval number: 1928). Patient data were collected in accordance with the Declaration of Helsinki and Ethical Guidelines for Medical and Health Research Involving Human Participants in Japan. The requirement for informed consent was waived by the ethics committee because the MSUS database was established as part of routine clinical practice.

## Results

Twenty patients with GCA–PMR and 40 with PMR were included in the analysis. Table 1 shows a summary of the clinical characteristics of the patients. The median age was 76.0 years in the GCA–PMR group and 74.5 in the PMR group, with no significant differences between the groups. No significant differences were found in the proportion of female patients. The median time from symptom onset to diagnosis was 90.0 days in the GCA–PMR group and 89.5 in the PMR group, with no significant differences between the groups. Furthermore, no significant differences were observed in the CRP level or ESR between the groups. Bilateral upper arm tenderness was more frequent in the PMR group than in the GCA–PMR group. According to the 2012 EULAR/ACR PMR classification criteria, excluding MSUS items, all patients in both groups exhibited morning stiffness and were negative for RF and anti-citrullinated protein antibodies.

**Table 1:** Baseline characteristics and PMR-related clinical features of patients with GCA–PMR and PMR.

	GCA–PMR	PMR	*P*-value
Number of patients, *n*	20	40	
Age, median [IQR], years	76.0 [72.5–83.5]	74.5 [65.5–77.0]	0.1023
Female, *n* (%)	16 (80)	27 (68)	0.3111
Time to diagnosis, median [IQR], days	90.0 [57.5–92.5]	89.5 [46.3–160.3]	0.6047
CRP, median [IQR], mg/dl	8.45 [3.87–9.98]	5.39 [2.07–9.52]	0.249
ESR, median [IQR], mm/h	101.0 [51.8–116.5]	90.5 [72.5–100.5]	0.2806
MMP-3, median [IQR], ng/ml	101.0 [37.1–422.1]	211.7 [135.7–375.1]	0.0501
Onset to maximal symptoms ≦2weeks, *n* (%)	5 (25)	21 (53)	0.0556
2012 EULAR/ACR classification criteria for PMR, score (no ultrasound), median [IQR]	5.0 [5.0–5.0]	5.0 [5.0–6.0]	0.123
Classified (score≧4), *n* (%)	20 (100)	40 (100)	1.000
Morning stiffness duration >45 min, *n* (%)	20 (100)	40 (100)	1.000
Hip pain or limited range of motion, *n* (%)	6 (30)	22 (55)	0.0999
Absence of RF or ACPA, *n* (%)	20 (100)	40 (100)	1.000
Absence of other joint pain, *n* (%)	12 (60)	25 (62)	1.000
Neck/shoulder gridle pain or limited range of motion, *n* (%)	15 (75)	35 (87)	0.2776
Bilateral shoulder pain, *n* (%)	20 (100)	40 (100)	1.000
Bilateral upper arm tenderness, *n* (%)	11 (55)	38 (95)	<0.001
Depression, *n* (%)	0 (0)	0 (0)	1.000

*P*-values were calculated by Mann–Whitney U test or Chi-square test and Fisher’s exact test.

IQR: interquartile range.

The clinical and imaging characteristics of patients with GCA–PMR are summarized in Table 2. Notably, all patients had LV-GCA, whereas cranial involvement was present in 50% of patients.

**Table 2:** Clinical characteristics of GCA in patients with GCA–PMR.

	GCA–PMR (*n* = 20)
2022 EULAR/ACR GCA classification criteria score, median [IQR]	9.0 [7.0–12.0]
Classified (score ≧6), *n* (%)	20 (100)
-Age ≧ 50 years at time of diagnosis	20 (100)
-Morning stiffness in shoulders/neck, *n* (%)	20 (100)
-Sudden visual loss, *n* (%)	0 (0)
-Visual impairment, *n* (%)	1 (5)
-Jaw or tongue claudication, *n* (%)	4 (20)
-New temporal headache, *n* (%)	6 (30)
-Scalp tenderness, *n* (%)	4 (20)
-Abnormal examination of the temporal artery, *n* (%)	1 (5)
-Maximum ESR ≥50 (mm/h) or maximum CRP ≥1 (mg/dl), *n* (%)	18 (90)
-Positive temporal artery biopsy, *n* (%)	0 (0)^a^
-Positive halo sign on artery ultrasound, *n* (%)	11 (55)
-Bilateral axillary involvement, *n* (%)	8 (40)
-FDG-PET activity throughout aorta, *n* (%)	12 (71)^a^
Vasculitis on contrast-enhanced CT, *n* (%)	17 (85)
Vasculitis on MRI, *n* (%)	5 (63)^a^
Cranial involvement only on imaging, *n* (%)	0 (0)
Large-vessel involvement only on imaging, *n* (%)	10 (50)
Both cranial and large-vessel involvement on imaging, *n* (%)	10 (50)
Clinically Silent GCA, *n* (%)	12 (60)

Silent GCA was defined as GCA diagnosed by imaging in patients who did not present with cranial or ischemic symptoms at the initial visit, including sudden visual loss, visual field defects, jaw or tongue claudication, limb claudication, new temporal headache, scalp tenderness or temporal artery abnormality on vascular examination.

a Percentages were calculated using the denominator of only patients who underwent each assessment (temporal artery biopsy, *n* = 3; FDG-PET, *n* = 17; MRI, *n* = 8).

FDG-PET: fluorodeoxyglucose-PET.

**Figure 1:**
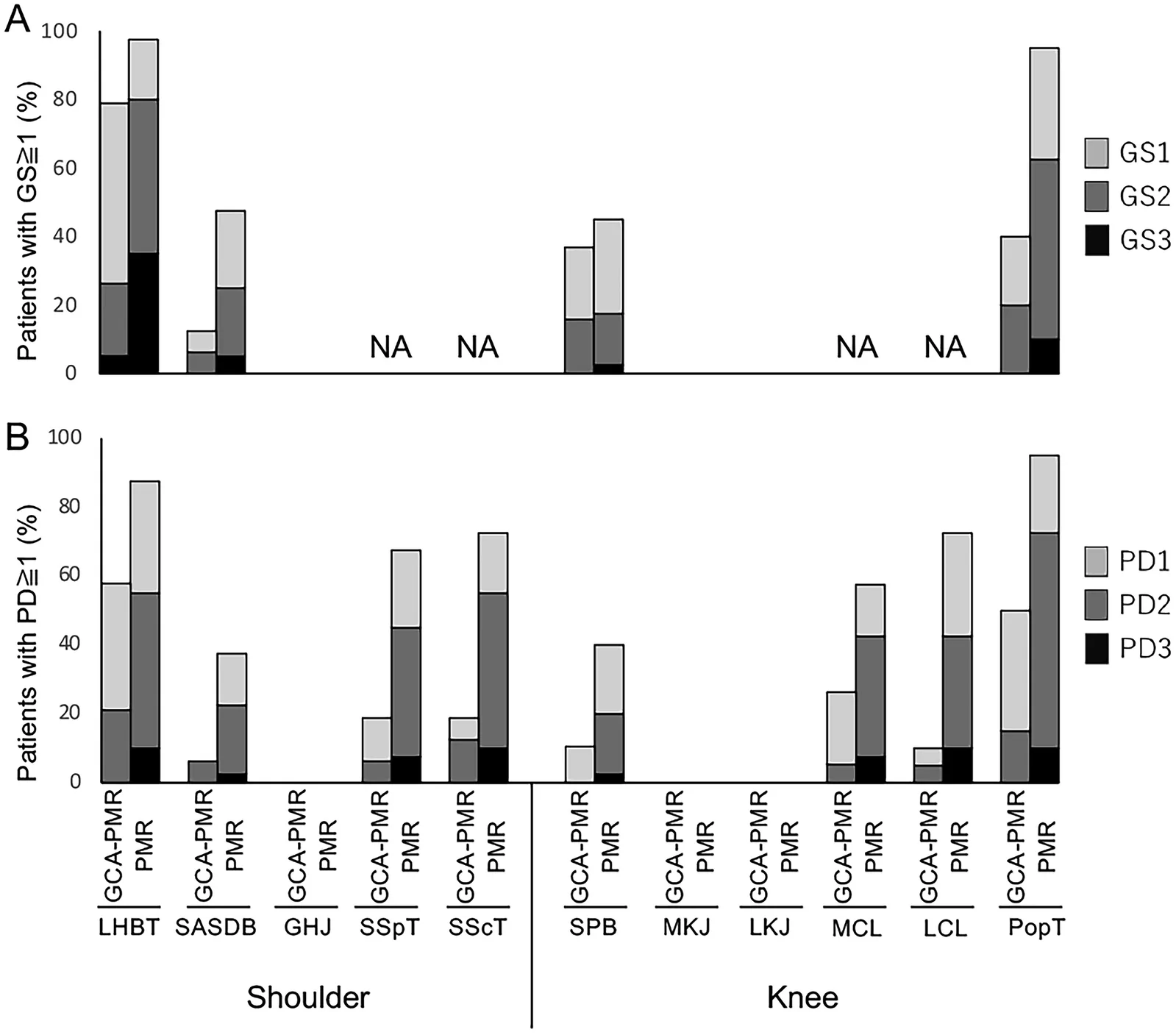
Prevalence of MSUS inflammatory findings in patients with GCA–PMR and PMR. Percentage of patients with positive ultrasound findings at each joint site (A: GS; B: PD). Stacked bars represent grades 1–3 and the sum of overall positive rates. Given that GS assessment is not defined for tendons, the SSpT, SScT, MCL, and LCL were denoted as NA. All sites were assessed bilaterally, and the higher grade between the left and right sides was adopted as the grade for each site. MSUS: musculoskeletal ultrasound; GS: greyscale; PD: power Doppler; NA: not applicable; LHBT: long head of the biceps tendon; SASDB: subacromial–subdeltoid bursa; GHJ: glenohumeral joint; SSpT: supraspinatus tendon; SScT: subscapularis tendon; SPB: suprapatellar bursa; MKJ: medial knee joint; LKJ: lateral knee joint; MCL: medial collateral ligament; LCL: lateral collateral ligament; PopT: popliteus tendon

Figure 1 illustrates the prevalence of MSUS-positive findings in the two groups. In both groups, GS abnormalities were predominantly observed in tenosynovitis, particularly involving the LHBT and PopT, whereas bursitis, including SASDB and SPB, was observed in approximately half of the patients. By contrast, intra-articular synovitis of the GHJ or knee joint was not observed. PD signals were detected not only at sites of GS abnormalities, but also in tendons (SSpT and SScT) and ligaments (MCL and LCL). However, the prevalence of GS and PD abnormalities was consistently lower in the GCA–PMR group than in the PMR group across all the assessed sites. Supplementary Table S1, available at *Rheumatology Advances in Practice* Online, presents a comparison of the median summed GS and PD scores for the left and right sides of each assessed site. For tenosynovitis (LHBT and PopT), tendinitis (SSpT and SScT) and ligamentitis (MCL and LCL), the GCA–PMR group exhibited lower scores than the PMR group across all sites and score combinations.

For tenosynovitis, tendinitis, ligamentitis and bursitis, which have a relatively high prevalence, the prevalence of lesions present on at least one side (either side) and the prevalence of bilateral lesions were compared between the two groups (Table 3). The definition of MSUS inflammatory findings used in the table was identical to that described in the Methods. Except for the bilateral involvement of the SASDB and SPB, the prevalence of all forms of tenosynovitis (LHBT and PopT), tendinitis (SSpT and SScT) and ligamentitis (MCL and LCL) was lower in the GCA–PMR group than in the PMR group. In analyses stratified by anatomical site and lesion category, the prevalence of all MSUS inflammatory findings remained consistently lower in the GCA–PMR group than in the PMR group.

**Table 3:** Site-level prevalence of musculoskeletal ultrasound findings in GCA–PMR and PMR.

	Present either unilaterally or bilaterally			Present bilaterally		
	GCA–PMR *n* = 20	PMR *n* = 40	*P*-value	GCA–PMR *n* = 20	PMR *n* = 40	*P*-value
Shoulder component, *n* (%)						
LHBT	12 (63)	39 (98)	<0.0001	4 (21)	34 (85)	<0.0001
SASDB	1 (6)	16 (40)	0.0054	1 (6)	6 (15)	0.2470
SSpT	3 (19)	27 (68)	<0.0001	2 (13)	15 (38)	0.0340
SScT	3 (19)	29 (73)	<0.0001	1 (6)	15 (38)	0.0229
Shoulder combination, *n* (%)						
Tendinitis (SSpT and/or SScT)	5 (26)	33 (83)	<0.0001	2 (13)	19 (48)	0.00431
Any (LHBT, SASDB, SSpT and/or SScT)	13 (68)	40 (100)	<0.0001	5 (26)	37 (93)	<0.0001
Knee component, *n* (%)						
SPB	4 (21)	16 (40)	0.1536	2 (11)	7 (18)	0.7040
MCL	5 (26)	23 (58)	0.0274	2 (11)	16 (40)	0.0189
LCL	2 (10)	29 (73)	<0.0001	1 (5)	17 (43)	0.0026
PopT	10 (50)	38 (95)	<0.0001	3 (15)	27 (68)	<0.0001
Knee combination, *n* (%)						
Ligament (MCL and/or LCL)	5 (25)	33 (83)	<0.0001	2 (10)	24 (60)	<0.0001
Any (SPB, MCL, LCL and/or PopT)	11 (55)	38 (95)	<0.0001	4 (20)	32 (80)	<0.0001
All combination, *n* (%)						
Any shoulder (LHBT, SASDB, SSpT, SScT) or any knee (SPB, MCL, LCL, PopT)	15 (75)	40 (100)	0.0028	7 (35)	39 (98)	<0.0001
Any shoulder (LHBT, SASDB, SSpT, SScT) and any knee (SPB, MCL, LCL, PopT)	9 (45)	38 (95)	<0.0001	2 (10)	30 (75)	<0.0001

‘Present either unilaterally or bilaterally’ indicates the presence of a positive finding in at least one of the left or right joints, whereas ‘present bilaterally’ indicates positive findings in both the joints.

Arthritis was considered positive when tenosynovitis (LHBT, PopT) or bursae (SASDB, SPB) showed a greyscale score ≥2 or a power Doppler score ≥1, and tendinitis (SSpT, SScT) and ligamentitis (MCL, LCL) showed a power Doppler score ≥1.

*P*-values were calculated by Chi-square test and Fisher’s exact test.

GS: greyscale; PD: power Doppler; LHBT: long head of the biceps tendon; SASDB: subacromial–subdeltoid bursa; SSpT: supraspinatus tendon; SScT: subscapularis tendon; MKJ: medial knee joint; LKJ: lateral knee joint; MCL: medial collateral ligament; LCL: lateral collateral ligament; PopT: popliteus tendon.

**Figure 2:**
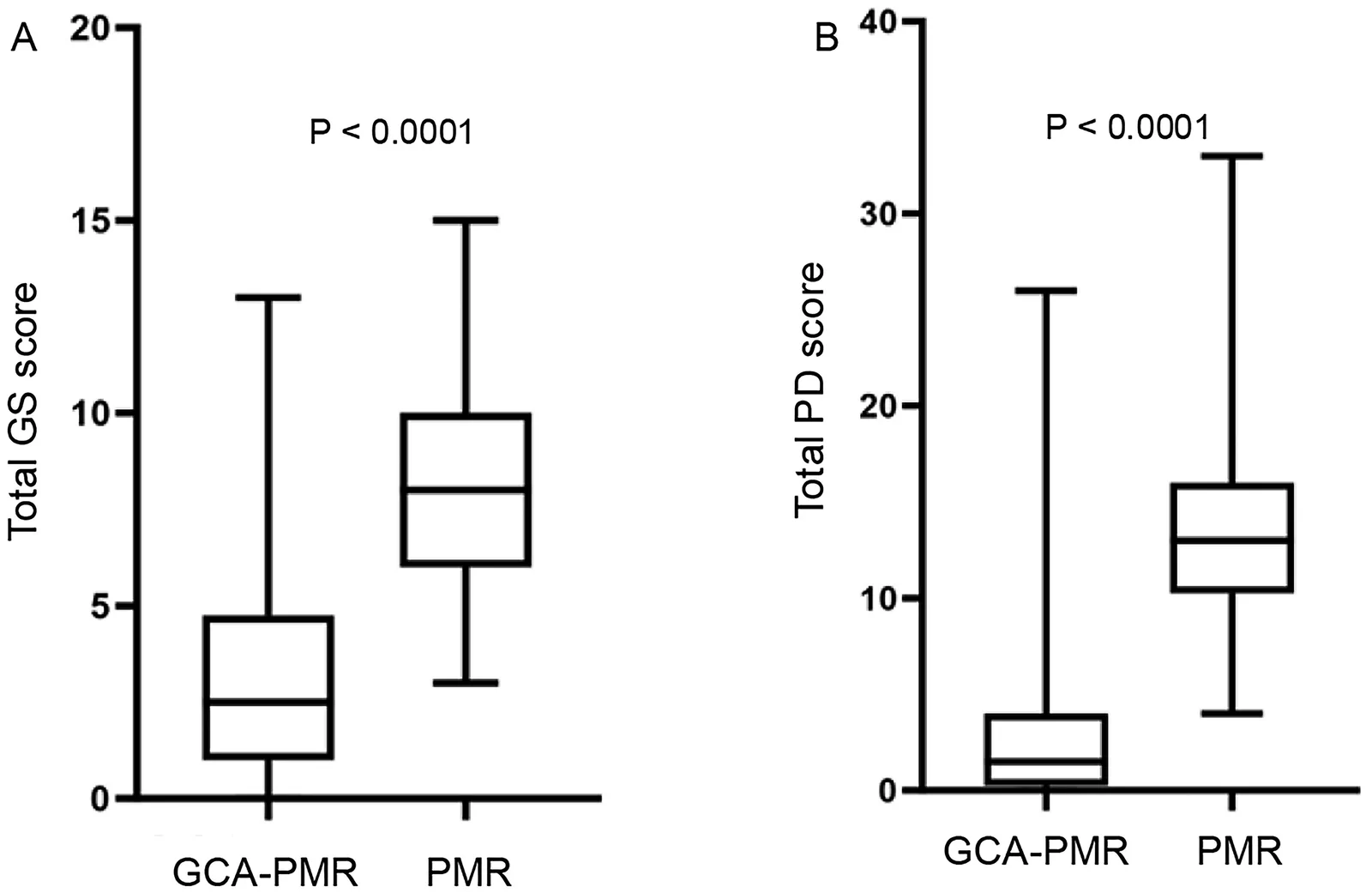
Comparison of total MSUS GS and PD scores between the GCA–PMR and PMR groups. *P*-values were calculated by the Mann–Whitney U test. (A) GS and (B) PD. The total GS score was defined as the sum of the GS scores (0–3) across seven assessed sites bilaterally (14 sites in total), excluding tendons (SSpT and SScT) and collateral ligaments (MCL and LCL), for which GS assessment was not performed; the maximum possible score was 42. The total PD score was defined as the sum of the PD scores (0–3) across all 11 assessed sites bilaterally (22 sites in total), with a maximum possible score of 66. MSUS: musculoskeletal ultrasound; GS: greyscale; PD: power Doppler; SSpT: supraspinatus tendon; SScT: subscapularis tendon; MCL: medial collateral ligament; LCL: lateral collateral ligament

Figure 2 shows a comparison of the total GS and PD scores between the two groups. Both total scores were significantly lower in the GCA–PMR group than in the PMR group, thus indicating that the site-specific differences observed in Fig. 1 and Supplementary Table S1, available at *Rheumatology Advances in Practice* Online, were consistently reflected in the composite measure. Collectively, these findings suggested that the overall musculoskeletal involvement on MSUS was lower in patients with GCA–PMR than in those with isolated PMR.

Supplementary Fig. S2, available at *Rheumatology Advances in Practice* Online, shows the distribution of serum CRP levels and total MSUS scores for all 60 patients. Across all three scoring patterns, namely, the total GS score (Supplementary Fig. S2A, available at *Rheumatology Advances in Practice* Online), total PD score (Supplementary Fig. S2B, available at *Rheumatology Advances in Practice* Online) and combined GS + PD score (Supplementary Fig. S2C, available at *Rheumatology Advances in Practice* Online), the GCA–PMR group exhibited lower MSUS-defined musculoskeletal involvement than the PMR group. By contrast, the distribution of CRP levels was comparable between the two groups. These findings suggest a dissociation between the musculoskeletal inflammatory burden and systemic inflammation in patients with GCA–PMR.

**Figure 3:**
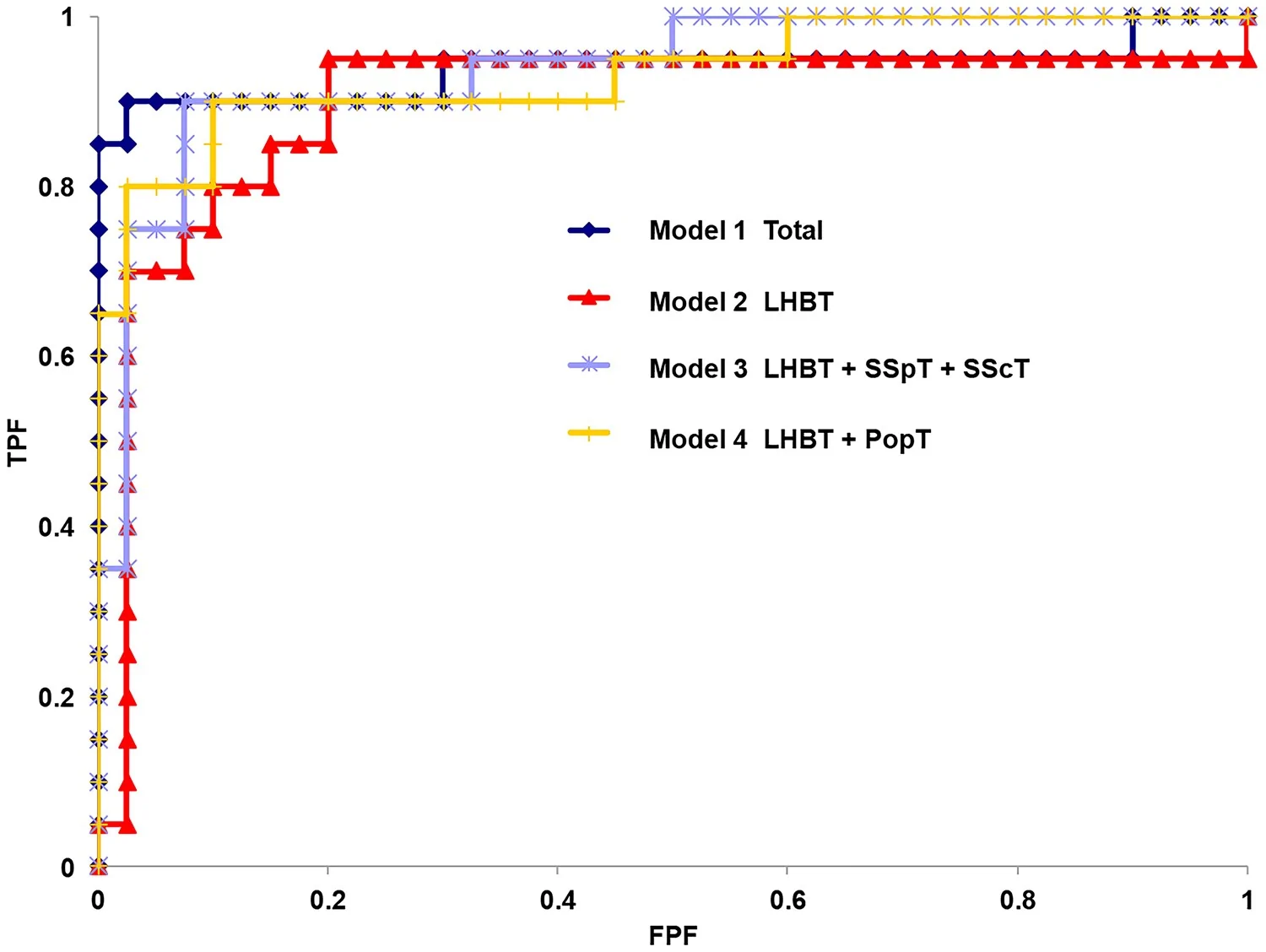
Receiver operating characteristic curves of the GCA–PMR MSUS prediction score. Model 1 (total score): GCA–PMR MSUS prediction score = 2.19 + 0.17 × CRP (mg/dl) − 0.26 × total GS + PD score. The AUC, cutoff value, sensitivity, specificity, PPV, and NPV were 0.94, 0.064, 0.9, 0.98, 0.95, and 0.95, respectively. Model 2 (LHBT): GCA–PMR MSUS prediction score = 1.24 + 0.17 × CRP (mg/dl) − 0.72 × GS + PD score in the bilateral LHBT. The AUC, cutoff value, sensitivity, specificity, PPV, and NPV were 0.9, −0.99, 0.95, 0.8, 0.69, and 0.97, respectively. Model 3 (LHBT + SSpT + SScT): GCA–PMR MSUS prediction score = 1.48 + 0.25 × CRP (mg/dl) − 0.62 × GS + PD score in the bilateral LHBT, SSpT, and SScT. The AUC, cutoff value, sensitivity, specificity, PPV, and NPV were 0.89, −1.08, 0.85, 0.93, 0.85, and 0.93, respectively. Model 4 (LHBT + PopT): GCA–PMR MSUS prediction score = 3.11 + 0.17 × CRP (mg/dl) − 0.63 × GS + PD score in the bilateral LHBT and PopT. The AUC, cutoff value, sensitivity, specificity, PPV, and NPV were 0.93, −0.47, 0.9, 0.9, 0.81, and 0.92, respectively. TPF: true-positive fraction; FPF: false-positive fraction; MSUS: musculoskeletal ultrasound; AUC: area under the curve; Sens: sensitivity; Spec: specificity; PPV: positive predictive value; NPV: negative predictive value; LHBT: long head of the biceps tendon; SSpT: supraspinatus tendon; SScT: subscapularis tendon; PopT: popliteus tendon

To explore whether a combination of serum CRP levels and MSUS scores could predict GCA in patients with PMR, an MSUS-based prediction model, called the GCA–PMR MSUS prediction score, was developed. The coefficients used in the prediction formulas were derived from a multivariable logistic regression analysis. Considering their feasibility in routine clinical practice and discriminatory performance, four MSUS score combinations were evaluated (Supplementary Table S2, available at *Rheumatology Advances in Practice* Online, Fig. 3). Among the four models, the prediction score incorporating all assessed sites (total GS + total PD score) achieved the highest AUC of 0.94. However, simplified models using limited anatomical sites, such as the LHBT plus PopT or the LHBT alone, also demonstrated acceptable discriminatory performance, thus suggesting that the assessment of a restricted number of sites may be sufficient for screening purposes in clinical practice.

## Discussion

This study compared shoulder and knee MSUS findings between patients with GCA presenting with PMR symptoms with those with isolated PMR. Across both the shoulder and knee joints, the frequency and severity of tenosynovitis (LHBT and PopT), tendinitis (SSpT and SScT) and ligamentitis (MCL and LCL) were consistently lower in patients with GCA–PMR than in those with isolated PMR. Collectively, these findings demonstrate that GCA–PMR is characterized by lower MSUS-defined musculoskeletal involvement than PMR. Consistent with previous reports, typical rheumatoid arthritis–like intra-articular synovitis was not observed in either group [6, 17]. A major strength of this study is that all imaging assessments were performed before treatment initiation, thereby minimizing the potential confounding effects of GC or immunosuppressive therapies and enhancing the reliability of the observed results.

Previous studies have addressed similar questions by using other imaging techniques. van der Geest *et al.* [8] used FDG-PET/CT to semi-quantitatively score tracer uptake at 30 anatomical sites, such as the shoulder girdle, pelvic girdle, knees and spine; they demonstrated that patients with GCA–PMR had lower scores than those with PMR. Similarly, Emamifar *et al.* [9] semi-quantitatively assessed FDG uptake in the shoulders, hips and spine of 64 patients with PMR and 10 with GCA–PMR; they showed that both the frequency and mean intensity of maximal FDG uptake were lower in the GCA–PMR group than in the PMR group. Studies using MRI have reported similar results. Kern *et al.* [10] retrospectively evaluated the inflammatory changes at nine shoulder sites on contrast-enhanced MRI, originally obtained to assess LVV. The proportion of patients with inflammatory findings in at least one site was 56% in the GCA–PMR group and 68% in the PMR group, thus indicating a lower frequency of inflammatory changes in patients with GCA than in those with PMR. By contrast, the mean number of inflamed sites per patient was higher in the GCA–PMR group than in the PMR group (3.1 vs. 2.6), thus suggesting a broader anatomical distribution of inflammatory involvement in GCA–PMR. These findings from other imaging modalities are consistent with our results, thus indicating that musculoskeletal involvement is less pronounced in patients with GCA presenting with PMR symptoms than in those with isolated PMR. To date, only one comparative study directly evaluated the MSUS findings in these two patient populations. Burg *et al.* [11] assessed 32 patients with PMR and 28 with GCA–PMR by using MSUS of the shoulders (LHBT, GHJ, and SASDB) and hips (trochanteric bursa effusion and hip joint capsule effusion) to quantify GS abnormalities on the basis of total area measurements; they reported that the total area of the hip joint capsule effusion was larger in the GCA–PMR group than in the PMR group. With regard to the MSUS findings, the discrepancy between their results and ours may be attributable to the small absolute differences in the measured areas, exclusive use of GS assessment, and differences in the anatomical sites evaluated, all of which may influence the interpretation of the inflammatory burden.

Differences in systemic inflammatory responses and cytokine profiles have been reported between GCA–PMR and PMR. Patients with GCA–PMR have lower serum interleukin-6 (IL-6) levels than those with PMR [18]. In PMR, biopsy specimens obtained from the shoulder bursae demonstrate abundant IL-6-positive cells [19, 20]. Similarly, serum MMP-3 levels are lower in patients with GCA–PMR than in those with PMR [20–22]. Although the mechanisms underlying these biomarker differences remain incompletely understood, our MSUS findings are consistent with previous observations demonstrating less musculoskeletal inflammation in GCA–PMR than in isolated PMR.

In this study, a GCA–PMR MSUS prediction score was also developed to estimate the presence of GCA in a group of patients with established PMR. Several prediction models for GCA have been reported; however, most have been designed for different clinical contexts. Laskou *et al.* [23] proposed the GCA probability score, which incorporates age, sex, clinical symptoms and signs, inflammatory markers, symptom duration and alternative diagnoses, as a clinical scoring system for patients with suspected GCA; their scoring system demonstrated a sensitivity of 95.7% and specificity of 86.7% for the diagnosis of GCA. Ing *et al.* [24] retrospectively analysed 688 patients who underwent temporal artery biopsy (TAB), developed a prediction model for TAB positivity, and identified age, jaw claudication, visual symptoms, platelet count and CRP level as key predictors. However, large-vessel GCA is less likely to be captured by TAB-based models, thus limiting applicability. A prediction model specifically targeting GCA with PMR symptoms was reported by Rodríguez-Valverde *et al.* [25], who retrospectively analysed 137 patients with isolated PMR and 90 patients with biopsy-proven GCA–PMR; they identified headache, jaw claudication and clinical abnormalities of the temporal artery as predictive factors. Notably, these models relied primarily on clinical features and laboratory data rather than imaging findings. To the best of our knowledge, no previous study has developed a prediction model on the basis of differences in MSUS findings between GCA–PMR and PMR. In our model, the contribution of CRP suggests that patients with PMR who have a lower MSUS-defined inflammatory burden despite comparable systemic inflammatory marker levels may be more likely to have GCA. This finding supports the concept that, in such cases, CRP levels may predominantly reflect vascular inflammation attributable to GCA rather than musculoskeletal inflammatory activity associated with PMR.

Currently, PMR and GCA are considered as part of a common spectrum disease (GCA–PMR spectrum disease, GPSD), due to the strict kinship represented by their numerous shared clinical and subclinical features. This perspective appears to be more appropriate than the traditional dichotomous approach to PMR and GCA in routine clinical practice [26–28]. Early diagnosis of GCA in patients with PMR before the onset of more specific vasculitis symptoms is required to potentially prevent irreversible vasculitis damage (e.g. permanent vision loss, stroke) as well as transient ischemic complications (e.g. limb claudication) [29]. Vascular imaging for GCA screening, though useful, may not be practical in every PMR patient [30, 31]. The GCA–PMR MSUS prediction score may be an alternative for selecting patients with PMR symptoms who warrant further evaluation for GCA. Although thorough MSUS of the shoulders and knees may be as time consuming as vascular ultrasound in clinical practice, a simplified MSUS assessment has demonstrated a similarly meaningful risk stratification, representing an important practical advantage of our approach.

This study had some important limitations. First, this was a single-centre study conducted at a university hospital in Japan with a relatively small sample size, which may have introduced selection and referral biases. Given that the study population consisted of referred patients, individuals with more severe manifestations of PMR may have been over-represented. Second, the study population comprised only Japanese patients. Ethnic differences in the clinical manifestations of PMR remain incompletely understood, and the prevalence and phenotype of GCA vary substantially across populations; therefore, the findings may not be directly generalizable to other ethnic groups. Third, systematic vascular imaging was not performed in all patients in the PMR group. Therefore, the possibility of subclinical GCA cannot be completely excluded. However, any such misclassification would likely have reduced the observed differences between the groups rather than exaggerated them. Fourth, although the MSUS assessment sites were selected in accordance with previous studies, the hip joints were not evaluated. Because ultrasound assessment of hip synovitis is technically more challenging than that of the shoulder or knee and has limited sensitivity for detecting deep-seated inflammatory lesions, the registry protocol focused on shoulder and knee involvement [32]. Nevertheless, hip involvement is a characteristic manifestation of PMR and is included in the 2012 EULAR/ACR provisional classification criteria. Therefore, the overall musculoskeletal inflammatory burden may not have been completely captured. Finally, the limited sample size precluded the external validation of the exploratory MSUS-based prediction model. Further studies in larger, independent cohorts are required to validate the performance and generalizability of this model.

## Conclusion

The study findings expand the understanding of GPSD by demonstrating that the MSUS-based assessment of the shoulder and knee joints, coupled with CRP levels, may support PMR patient selection for GCA screening. Future larger, multicentre and multiethnic prospective studies are warranted to validate these findings and establish their generalizability and clinical applicability.

## Supplementary Material

rkag110_Supplementary_Data

## Data Availability

The data underlying this article are not publicly available due to privacy and ethical restrictions. The data are available from the corresponding author upon reasonable request.
