# Supplementary material for: Musculoskeletal ultrasound findings in GCA with PMR and isolated PMR
Source: Rheumatol Adv Pract. 2026 Sep 12;10(4):rkag110. doi: 10.1093/rap/rkag110 (PMC13615851; doi:10.1093/rap/rkag110)
Supplement: Supplementary file 1 [file rkag110_supplementary_data.docx]

Supplementary Table S1. Comparison of the median sums of the left- and right-sided grayscale and power Doppler scores at each assessed site on musculoskeletal ultrasound.

|  |  | GCA–PMR n=20 | PMR n=40 | P-value |
| --- | --- | --- | --- | --- |
| Shoulder component |  |  |  |  |
| LHBT, median [IQR] | GS | 1 [0.75 - 2] | 4 [3 - 5] | <0.001 |
|  | PD | 1 [0 - 2] | 3 [1 - 4] | <0.001 |
|  | Total | 2 [1.5 - 3.25] | 6 [5 - 8] | 0.0035 |
| SASDB, median [IQR] | GS | 0 [0 - 0] | 0 [0 - 2] | 0.007 |
|  | PD | 0 [0 - 0] | 0 [0 - 1.25] | 0.127 |
|  | Total | 0 [0 - 0] | 0.5 [0 - 3] | 0.0041 |
| GHJ, median [IQR] | GS | 0 [0 - 0] | 0 [0 - 0] | 1.0 |
|  | PD | 0 [0 - 0] | 0 [0 -0] | 1.0 |
|  | Total | 0 [0 - 0] | 0 [0 - 0] | 1.0 |
| SSpT, median [IQR] | PD | 0 [0 - 0] | 2 [0 - 3] | <0.001 |
|  | Total | 0 [0 - 0] | 2 [0 - 3] | <0.001 |
| SScT, median [IQR] | PD | 0 [0 - 0] | 2 [0 - 3] | <0.001 |
|  | Total | 0 [0 - 0] | 2 [0 - 3] | <0.001 |
| Shoulder combination, median [IQR] | GS | 1 [0.75 - 2] | 4 [3 - 6] | <0.001 |
|  | PD | 1 [0 - 2] | 7 [4 - 9] | <0.001 |
|  | Total | 2 [1.75 - 4] | 12 [8 - 14] | <0.001 |
| Knee component |  |  |  |  |
| SPB, median [IQR] | GS | 0 [0 - 2] | 0 [0 - 2] | 0.6988 |
|  | PD | 0 [0 - 0] | 0 [0 - 1.25] | 0.0216 |
|  | Total | 0 [0 - 2] | 0 [0 - 3] | 0.3481 |
| MKJ, median [IQR] | GS | 0 [0 - 0] | 0 [0 - 0] | 1.0 |
|  | PD | 0 [0 - 0] | 0 [0 - 0] | 1.0 |
|  | Total | 0 [0 - 0] | 0 [0 - 0] | 1.0 |
| LKJ, median [IQR] | GS | 0 [0 - 0] | 0 [0 - 0] | 1.0 |
|  | PD | 0 [0 - 0] | 0 [0 - 0] | 1.0 |
|  | Total | 0 [0 - 0] | 0 [0 - 0] | 1.0 |
| MCL, median [IQR] | PD | 0 [0 - 0.25] | 1.5 [0 - 3] | 0.0075 |
|  | Total | 0 [0 - 0.25] | 1.5 [0 - 3] | 0.0075 |
| LCL, median [IQR] | PD | 0 [0 - 0] | 2 [0 - 3] | <0.001 |
|  | Total | 0 [0 - 0] | 2 [0 - 3] | <0.001 |
| PopT, median [IQR] | GS | 0 [0 - 1.25] | 3 [2 - 3] | <0.001 |
|  | PD | 0.5 [0 - 1] | 2.5 [2 - 4] | <0.001 |
|  | Total | 0.5 [0 - 3] | 5.5 [4 - 7] | <0.001 |
| Knee combination, median [IQR] | GS | 1.5 [0 - 2.25] | 3 [2 - 5] | <0.001 |
|  | PD | 0.5 [0 - 2] | 6.5 [4 - 9] | <0.001 |
|  | Total | 2.5 [0 - 4.25] | 10 [6.75 - 13] | <0.001 |
| LHBT+PopT, median [IQR] | GS | 2 [0.75 - 3.25] | 4 [4 - 6] | <0.001 |
|  | PD | 1 [0 - 2] | 3 [2 - 4.25] | <0.001 |
|  | Total | 2.5 [1.5 - 5] | 7 [6 - 10] | <0.001 |
| LHBT+SSpT+SScT, median [IQR] | GS | 1 [0.75 - 2] | 4 [3 - 5] | <0.001 |
|  | PD | 1 [0 - 2] | 6 [3.75 - 8.25] | <0.001 |
|  | Total | 2 [1.75 - 4] | 10 [7.75 - 13] | <0.001 |
| MCL+LCL+PopT, median [IQR] | GS | 0 [0 - 1.25] | 3 [2 - 3] | <0.001 |
|  | PD | 0.5 [0 - 2] | 6 [3.75 - 7.25] | <0.001 |
|  | Total | 1 [0 - 3] | 8.5 [5.75 - 11] | <0.001 |
| All combination, median [IQR] | GS | 2.5 [1 - 4.25] | 8 [6 - 10] | <0.001 |
|  | PD | 1.5 [0.75 - 4] | 13 [10.75 - 16] | <0.001 |
|  | Total | 5 [2 - 7.5] | 21.5 [17 - 24.25] | <0.001 |

P-values were calculated by Mann–Whitney U test.

For each site, the GS score represented the sum of the left- and right-sided grayscale scores (maximum 6), the PD score represented the sum of the left- and right-sided power Doppler scores (maximum 6), and the total score represented the sum of the GS and PD scores (maximum 12).

Abbreviations: GS, grayscale; PD, power Doppler; NA, not applicable; LHBT, long head of the biceps tendon; SASDB, subacromial–subdeltoid bursa; GHJ, glenohumeral joint; SSpT, supraspinatus tendon; SScT, subscapularis tendon; SPB, suprapatellar bursa; MKJ, medial knee joint; LKJ, lateral knee joint; MCL, medial collateral ligament; LCL, lateral collateral ligament; PopT, popliteus tendon.

Supplementary Table S2. Performance of the GCA–PMR MSUS prediction score

| Model | Prediction formula | AUC | Cut-off | Sens | Spec | PPV | NPV |
| --- | --- | --- | --- | --- | --- | --- | --- |
| Total | 2.19 + 0.17 × CRP (mg/dL) − 0.26 × total GS plus PD score* | 0.94 | 0.06 | 0.9 | 0.98 | 0.95 | 0.95 |
| LHBT | 1.24 + 0.17 × CRP (mg/dL) − 0.72 × GS plus PD score in the bilateral LHBT^†^ | 0.9 | -0.99 | 0.95 | 0.8 | 0.69 | 0.97 |
| LHBT/SSpT/SScT | 1.48 + 0.25 × CRP (mg/dL) − 0.62 × GS plus PD score in the bilateral LHBT, SSpT, and SScT^‡^ | 0.89 | -1.08 | 0.85 | 0.93 | 0.85 | 0.93 |
| LHBT/PopT | 3.11 + 0.17 × CRP (mg/dL) − 0.63 × GS plus PD score in the bilateral LHBT and PopT^§^ | 0.93 | -0.47 | 0.9 | 0.9 | 0.81 | 0.92 |

* Total GS plus PD score, calculated as the sum of GS and PD scores across all assessed sites (maximum possible score, 108).

† GS plus PD score in the bilateral LHBT (maximum possible score, 12).

‡ GS plus PD score in the bilateral LHBT, SSpT, and SScT (maximum possible score, 24).

§ GS plus PD score in the bilateral LHBT and PopT (maximum score, 24).

The coefficients used in the prediction formulas were derived from multivariable binary logistic regression analysis. The presence or absence of concomitant GCA was used as the dependent variable, and CRP levels and MSUS scores were considered independent variables.

Abbreviations: MSUS, musculoskeletal ultrasound; area under the curve, AUC; Sens, sensitivity; Spec, specificity; PPV, positive predictive value; NPV, negative predictive value;

LHBT, long head of the biceps tendon; SSpT, supraspinatus tendon; SScT, subscapularis tendon; PopT, popliteus tendon;

**Supplementary Figure S1. Flowchart of patient selection for the GCA–PMR group.** Patients were identified from a prospectively maintained musculoskeletal ultrasound (MSUS) registry of patients with polymyalgia rheumatica (PMR). Patients without a clinical diagnosis of giant cell arteritis (GCA), those who had received treatment before referral, or those without an adequate MSUS assessment were excluded. All included patients fulfilled both the 2022 ACR/EULAR classification criteria for GCA and the 2012 EULAR/ACR provisional classification criteria for PMR. Abbreviations: GCA, giant cell arteritis; MSUS, musculoskeletal ultrasound; PMR, polymyalgia rheumatica.

**Supplementary Figure 2. Scatter plots showing CRP and total MSUS scores in the GCA–PMR and PMR groups.** (A–C) Scatter plots of ultrasound composite scores in relation to serum CRP levels in the two patient groups (GCA–PMR [n = 20] and PMR [n = 40]). (A) Total GS scores versus CRP levels. (B) Total PD scores versus CRP levels. (C) Combined GS + PD scores versus CRP levels. The total GS score was defined as the sum of the GS scores (0–3) across seven assessed sites bilaterally (14 sites in total), excluding tendons (SSpT, SScT) and collateral ligaments (MCL, LCL), for which GS assessment was not performed; the maximum possible score was 42. The total PD score was defined as the sum of the PD scores (0–3) across all 11 assessed sites bilaterally (22 sites in total), with a maximum possible score of 66. The maximum possible value of the combined GS and PD scores was 108. Abbreviations: CRP, C-reactive protein; MSUS, musculoskeletal ultrasound; PMR, polymyalgia rheumatica; GCA, giant cell arteritis; GS, grayscale; PD, power Doppler.
